# Insights into Reston virus spillovers and adaption from virus whole genome sequences

**DOI:** 10.1371/journal.pone.0178224

**Published:** 2017-05-25

**Authors:** César G. Albariño, Lisa Wiggleton Guerrero, Harley M. Jenks, Ayan K. Chakrabarti, Thomas G. Ksiazek, Pierre E. Rollin, Stuart T. Nichol

**Affiliations:** 1 Centers for Disease Control and Prevention, Atlanta, GA, United States of America; 2 University of Texas Medical Branch, Galveston, TX, United States of America; Division of Clinical Research, UNITED STATES

## Abstract

Reston virus (family *Filoviridae*) is unique among the viruses of the *Ebolavirus* genus in that it is considered non-pathogenic in humans, in contrast to the other members which are highly virulent. The virus has however, been associated with several outbreaks of highly lethal hemorrhagic fever in non-human primates (NHPs), specifically cynomolgus monkeys (*Macaca fascicularis*) originating in the Philippines. In addition, Reston virus has been isolated from domestic pigs in the Philippines. To better understand virus spillover events and potential adaption to new hosts, the whole genome sequences of representative Reston virus isolates were obtained using a next generation sequencing (NGS) approach and comparative genomic analysis and virus fitness analyses were performed. Nine virus genome sequences were completed for novel and previously described isolates obtained from a variety of hosts including a human case, non-human primates and pigs. Results of phylogenetic analysis of the sequence differences are consistent with multiple independent introductions of RESTV from a still unknown natural reservoir into non-human primates and swine farming operations. No consistent virus genetic markers were found specific for viruses associated with primate or pig infections, but similar to what had been seen with some Ebola viruses detected in the large Western Africa outbreak in 2014–2016, a truncated version of VP30 was identified in a subgroup of Reston viruses obtained from an outbreak in pigs 2008–2009. Finally, the genetic comparison of two closely related viruses, one isolated from a human case and one from an NHP, showed amino acid differences in the viral polymerase and detectable differences were found in competitive growth assays on human and NHP cell lines.

## Introduction

Six members of the family *Filoviridae*, Ebola (EBOV), Sudan (SUDV), Bundibugyo, Marburg (MARV), Ravn (RAVV), and Taï Forest viruses, cause severe, often fatal disease in humans and have been responsible for sporadic outbreaks of hemorrhagic fevers on the African continent. In contrast, Reston (RESTV) and Lloviu (LLOV) viruses, have not been associated with human disease to date [1–3]. In particular, RESTV causes fatal disease in cynomolgus monkeys (*Macaca fascicularis*), and was originally identified in 2 separate groups of these animals that were imported from the Philippines into the United States in 1989. The first shipment of animals was received at two research facilities in Reston, Virginia (USA) and Philadelphia, Pennsylvania (USA) in October and November of 1989, respectively. Shortly after these facilities were depopulated and carefully disinfected, two additional shipments of animals were received at the Reston research facility and another in Alice, Texas (USA) in January and February of 1990, respectively. RESTV was detected again in the animals housed within these 2 facilities [4–7].

RESTV was later found on two additional occasions when cynomolgus monkeys were imported from the Philippines to research facilities in Sienna (Italy) and in Alice, Texas, (USA) in 1992 and 1996, respectively [8, 9]. More recently, RESTV was detected in domestic pigs (*Sus scrofa*) in the Philippines in 2008 and 2009, and evidence of RESTV infection was reported in pigs in China in 2011. Interestingly, no clear signs of an Ebolavirus-like disease were observed in these pigs [10, 11].

During the past few decades, full-length genome sequences have become available for most known filoviruses, and meticulous phylogenetic analyses has allowed the inference of their genetic relationships and their possible evolutionary origins [12–14]. In particular, facilitated by the use of high-throughput next-generation sequencing (NGS) technologies, an extensive collection of full-length genomic sequences of EBOV [15–17] was obtained during the 2013–2016 outbreak of Ebola virus disease (EVD) which involved ~28,000 confirmed cases and more than 11,000 deaths in Western Africa [18].

In contrast to EBOV, only a limited number of full-length genome sequences is available for RESTV [10, 12, 19, 20]. In this regard, and to the best of our knowledge, most studies on RESTV, including the interferon (IFN) antagonistic activities of viral proteins, transcription and replication factors, and virulence assessment in animal models were based on the reference strain that was isolated from cynomolgus monkeys in the USA in 1989 (GenBank # NC_004161). In order to expand the limited number of available full-length sequences of RESTV, we used NGS technology to determine the complete genomic sequence of 9 viral isolates that were obtained between 1989 and 2009 from a variety of hosts including a human case, non-human primates and domestic pigs. Five of these genomes correspond to novel viral isolates, while four of them were previously sequenced using traditional methods.

This report provides a better understanding on the diversity of RESTV, and will facilitate further studies aimed to address the pathogenicity of this virus and the initial spillovers events from the still unknown natural host into the human population.

## Materials and methods

### Cell culture and biosafety

All work with infectious viruses was performed in a biosafety level 4 (BSL-4) facility. RESTV isolates were propagated in Vero-E6 cells. These cells were propagated in Dulbecco’s modified Eagle’s medium (DMEM) supplemented with 5% fetal bovine serum and penicillin-streptomycin. Virus titers were determined by tissue culture infective dose 50 (TCID_50_) assays as previously described [21].

### Viruses

Available information of viruses used in this study is listed in Table 1, including the isolate name, collection date, GenBank accession number, source of the isolate, passage history, and literature references.

**Table 1:** Sequences analyzed in this study.

Isolate name	Collection date	GenBank Acc.no.	Source of viral isolates	Reference
**USA_PA_1989 (813159)**	**1989**	**KY798004**	**Primate imported from Philippines to Pennsylvania, USA. Virus isolated from liver of dead monkey (M. fascicularis). Animal # A15CY2, received in the Buckshire corporation in Perkasie, Pennsylvania; part of a shipment of 50 monkeys. Two passages in Vero E6 cells**	*
**USA_PA_1989**	**1989**	**NC_004161**	**Same isolate as above**	**Groseth et al, 2002**
**USA_VA_1989 (813168)**	**1989**	**KY798005**	**Primate imported from Philippines to Virginia, USA. Virus isolated from serum of dead monkey (M. fascicularis). Animal #53, room F. Two passages in Vero E6 cells**	*
**USA_VA_1989 (811952)**	**1989**	**KY798006**	**42 year old man that suffer laceration of the left index while performing necropsy on nonhuman primate (M. fascicularis) in Virginia, USA. Virus isolated from blood on day 11 post-contamination. The patient remained asymptomatic and developed Ebola antibodies by day 16 post-inoculation. One passage in CV7 cells**	*
**PHL_1989**	**1989**	**KY008770**	**Primate sample collected in Philippines. Virus isolated (89-AZ-1435) from spleen of a monkey (M. fascicularis) with signs of respiratory infection. One passage in MA-104 and 2 passages in Vero E6 cells**	**Cornish et al, 2017**
**ITA_1992 (806679)**	**1992**	**KY798007**	**Primate imported from Philippines to Sienna, Italy. Virus isolated from serum of M. fascicularis. Animal # 12559. Two passages in Vero E6 cells**	*
**PHL_1992 (806676)**	**1992**	**KY798008**	**Primate sample collected in Philippines. Virus isolated from liver of M. fascicularis. Two passages in Vero E6 cells**	*
**USA_TX_1996 (807334)**	**1996**	**KY798009**	**Primate imported from Philippines to Alice, Texas, USA. Virus isolated from liver of dead monkey (M. fascicularis). Animal # MkCQ8167 (10–1), room 8 #2. One passage in Vero E6 cells**	*
**USA_TX_1996**	**1996**	**JX477166**	**Same isolate as above**	**Carroll et al, 2013**
**PHL_A_2008 (811411)**	**2008**	**KY798010**	**Pig sample collected on farm A, Philippines. Virus isolated from lung tissue collected in Sto Nino, Bulacan on June 4–2008. Plum Island isolate #14595, harvested 11dpi. One passage in Vero E6 cells**	*
**PHL_A_2008**	**2008**	**FJ621583**	**Same isolate as above**	**Barrette et al, 2009**
**PHL_A_2008 (811412)**	**2008**	**KY798011**	**Pig sample collected on farm A, Philippines. Virus isolated from lymph node tissue collected in Sto Nino, Bulacan on June 4–2008. Plum Island isolate #14597, harvested 9dpi. One passage in Vero E6 cells**	*
**PHL_C_2008**	**2008**	**FJ621584**	**Pig sample collected on farm C, Philippines. Virus isolated from lymph node. One passage in Vero E6 cells**	**Barrette et al, 2009**
**PHL_E_2008**	**2008**	**FJ621585**	**Pig sample collected on farm E, Philippines. Virus isolated from spleen tissue. One passage in Vero E6 cells**	**Barrette et al, 2009**
**PHL_A_2009 (813161)**	**2009**	**KY798012**	**Pig sample collected on farm A, Philippines. Virus isolated from lung tissue collected in Sto Nino, Bulacan on Jan 8, 2009. Plum Island isolate #7376, animal # 149. One passage in Vero E6 cells**	*
**PHL_A_2009**	**2009**	**JX477165**	**Same isolate as above**	**Carroll et al, 2013**

* Sequences determined in this study

### Sequence analysis

Total RNA from cell culture supernatants was used for NGS. cDNA libraries were constructed using TruSeq Stranded mRNA kit (Illumina), following a modified version of the manufacturer’s protocol that skipped steps necessary for mRNA purification and for rRNA depletion. Sequencing was performed using the paired-end 2 × 150 chemistry on an Illumina MiniSeq instrument. Nucleotide and aa sequence alignments were generated using CLC Genomics Workbench 9.1 (www.clcbio.com). The same software package was used to perform the analysis of next-generation sequencing data, including read mapping, contig assembly, and variant detection. Additional sequencing details including the number of mapped reads, the length of the consensus sequence, % coverage of reference sequence, the number of ambiguous or missing nt at the termini, and the average coverage, are shown in S1 Table. The raw data will be available upon request.

### Phylogenetic analysis

Sequence alignments of complete genomes were used to perform a phylogenetic analysis with a Bayesian algorithm (Mr.Bayes) using Geneious 10.0.5 (www.geneious.com) using the following parameters: GTR substitution model, chain length of 1,100,000 generations, subsampling frequency of 200, burning length of 100,000, and random seed of 568. An additional Bayesian analysis including SUDV as outgroup is shown in S1 Fig. The complete genome alignments were also used to perform a Maximum-Likelihood phylogenetic analysis (S2 Fig) using default parameters in CLC Genomics Workbench 9.1 (www.clcbio.com).

### Competitive fitness assay

To assess the relative viral fitness of the human-derived virus isolate (USA_VA_1989, 811952) and the closest related virus isolated from a dead monkey (USA_VA_1989, 813168), the 2 viruses were mixed in an approximate 60:40% ratio, based on respective titers. The mix was used to infect VeroE6 cells (African green monkey) using a moi of 0.1. An additional experiment was performed by infecting human-derived Huh7 cells with the same viruses in an approximate ratio of 70:30%. After 7 days post infection, cell supernatants were collected, clarified by low-speed centrifugation, diluted 1:10, and used to infect fresh Vero E6 cells. A total of 3 passages were performed over 21 days, while aliquots of undiluted cell supernatants were also collected for RNA extraction using standard protocols. In order to minimize the possibility of random events during the initial infection, 3 independent infections were performed and subjected to the same passaging protocol. Total RNA from cell supernatants was used for next-generation sequencing as explained above. The variant detection analysis, including the number of reads, the coverage, and the frequency of nucleotide changes of the fitness assay is shown in S2 Table.

## Results and discussion

A complete list of the virus isolates and sequences analyzed in this study is provided in Table 1, including all available information, such as collection date, source of the isolate, passage history, GenBank accession numbers and literature references. In particular, we determined the genomic sequences of virus isolates obtained in 1989 from two primates housed in research facilities in Pennsylvania and Virginia, USA. In addition, we sequenced the genome of the only known virus isolate obtained from a human in 1989. This individual’s infection resulted from accidental exposure due to a wound of an animal handler performing a necropsy on an infected non-human primate. According to the records of this event [22], this individual and two additional animal handlers had detectable antibody but did not suffer from fever or other overt clinical signs or symptoms.

In addition, we sequenced the viral genomes of two isolates obtained in 1992: one isolated from a monkey imported from a breeding facility in the Philippines to Italy, and a second, obtained from a monkey that was housed in the same breeding facility [8]. In contrast to the 1989 incident, none of the animal handlers working in this facility developed a detectable antibody response nor showed clinical symptoms [23].

Finally, we also used NGS technology to confirm the genomic sequence of four virus isolates that were previously determined using the traditional sequencing (Sanger) method by our group and others. One of these viruses was isolated from a monkey imported from the Philippines to Texas in 1996 [9, 12]; the other 3 isolates were obtained from pigs during the 2008–2009 investigation of large farming operations in the Philippines [10, 12].

**Fig 1:**
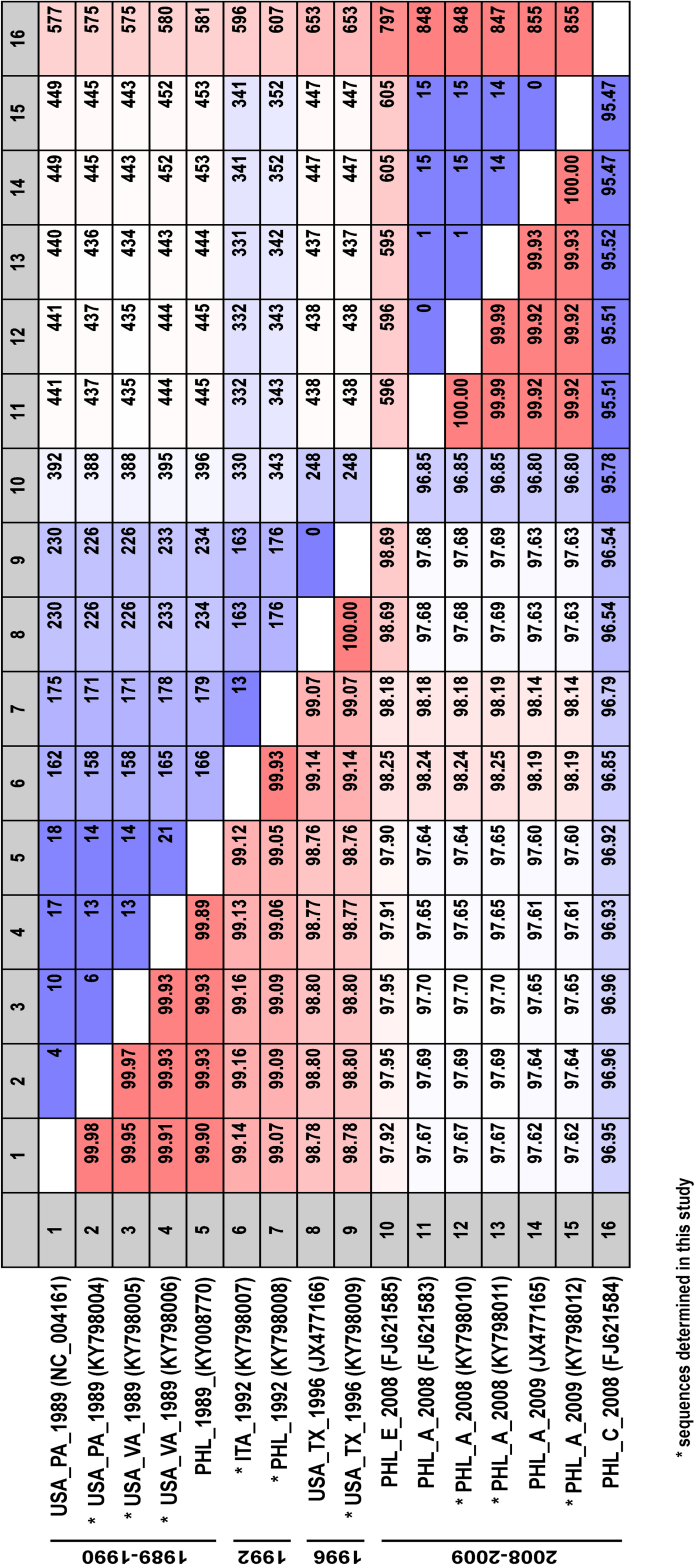
Sequence comparison of full-length viral genomes of representative RESTV isolates. Sequence identity (%) is indicated in the lower diagonal half, while the number of differing residues is indicated in the upper diagonal half. Asterisks indicate the 9 sequences determined in this study.

As shown in Fig 1, the comparison of the full-length RESTV genome shows a high degree of sequence identity (>95%) between all virus isolates obtained from either primates, pigs or the human case. As expected, the high identity of RESTV genomes translates into a low diversity in the amino acid (aa) sequences of each viral protein: <2% for VP35, VP24, and L; <5% for NP, VP40, and VP30; <10% for GP. Regarding the latter protein, the mucin-like domain (aa 313–501) of RESTV GP accounts for the highest diversity (<20%), a feature which is well known in the glycoprotein of all other filoviruses [2].

**Fig 2:**
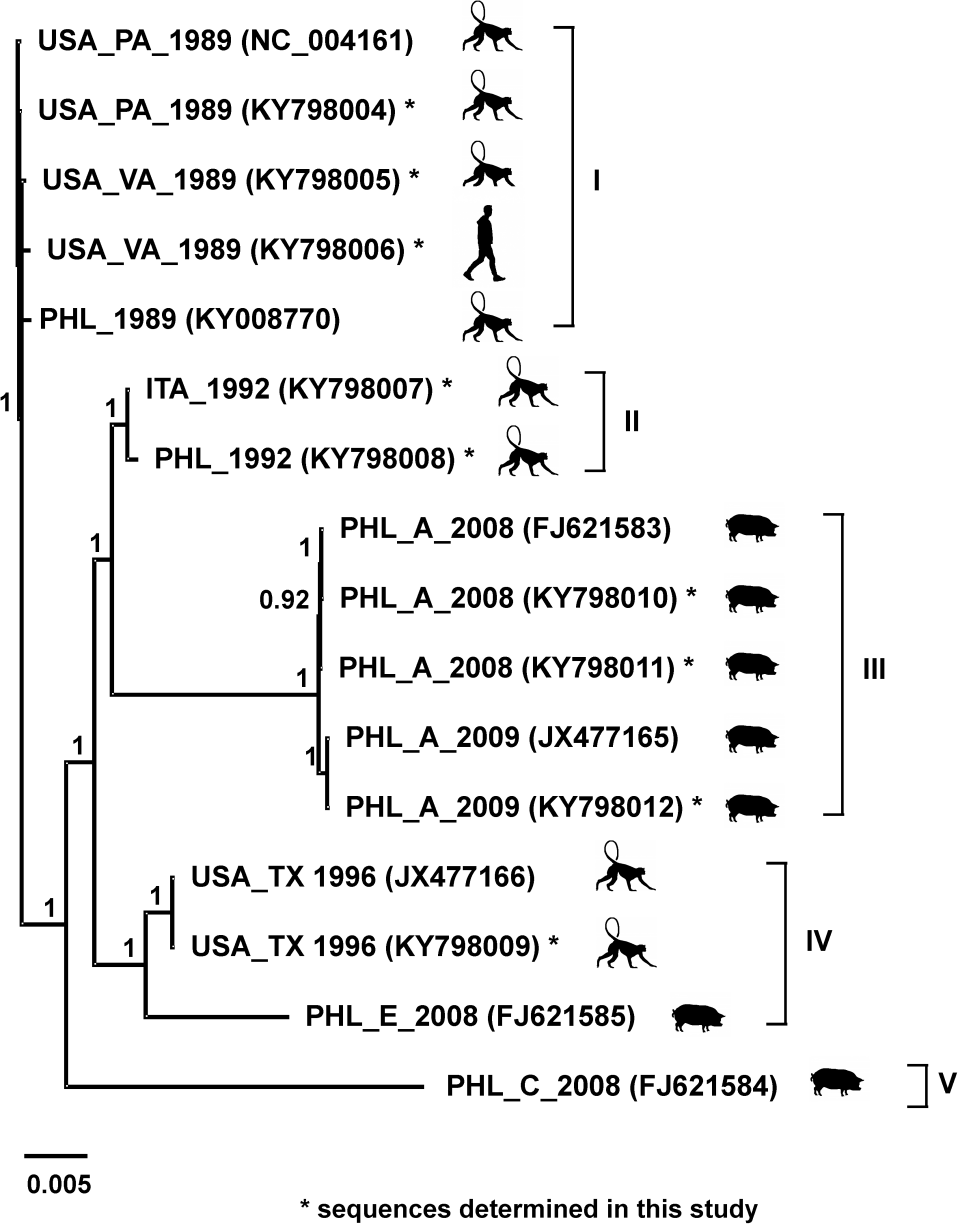
Phylogenetic analysis of available full-length RESTV genomes. Phylogenetic analysis was done with a Bayesian algorithm using Geneious 6.1.5. Clades I to V are indicated with brackets, Asterisks indicate the 9 sequences determined in this study. Posterior probabilities are listed next to each nodes. Scale bar indicates nucleotide substitutions per site.

Phylogenetic analysis (Fig 2) shows the 16 RESTV genomic sequences (obtained from 12 viral isolates) distributed into five lineages with strong support (posterior probability >0.9). Lineage I included all isolates obtained in 1989–1990 from the USA and the Philippines, while lineage II included the isolates obtained in 1992 from Italy and the Philippines. As described in 2 previous reports [10, 12], RESTV was isolated from different samples during the investigation of swine operations in the Philippines in 2008–2009. These samples were obtained from animals housed at different farms (Farms A, C, and E), exhibited relatively high diversity. In consequence, their corresponding genomes are distributed into different lineages in our phylogenetic analysis. For example, all viruses isolated from pigs housed on farm A (PHL_A_2008 and PHL_A_2009) are included in lineage III; while the isolate from a pig housed on farm E (PHL_E_2008) is grouped in lineage IV together with the isolates obtained from monkeys in 1996 (USA_TX_1996). Moreover, the most divergent isolate analyzed in this report was obtained from a pig housed on farm C (PHL_C_2008) and is uniquely placed in lineage V.

Overall, phylogenetic analysis of the RESTV isolates indicates a lack of temporal or geographic clustering. For example, the viruses isolated from monkeys in 1992 (lineage II) are more closely related to viruses isolated from pigs in 2008–2009 (lineage III) than to viruses isolated from monkeys in 1989–1990 and 1996 (lineages I and IV, respectively). The results of this analysis suggest at least 3 independent RESTV introduction events occurred from the unknown natural reservoir into the NHP breeding facilities in the Philippines. Similarly, the large diversity of the viruses isolated from pigs during the 2008–2009 investigation, which are grouped into 3 different lineages, also suggests the possibility of at least 3 independent introduction events from the natural reservoir into the different pig farms.

**Fig 3:**
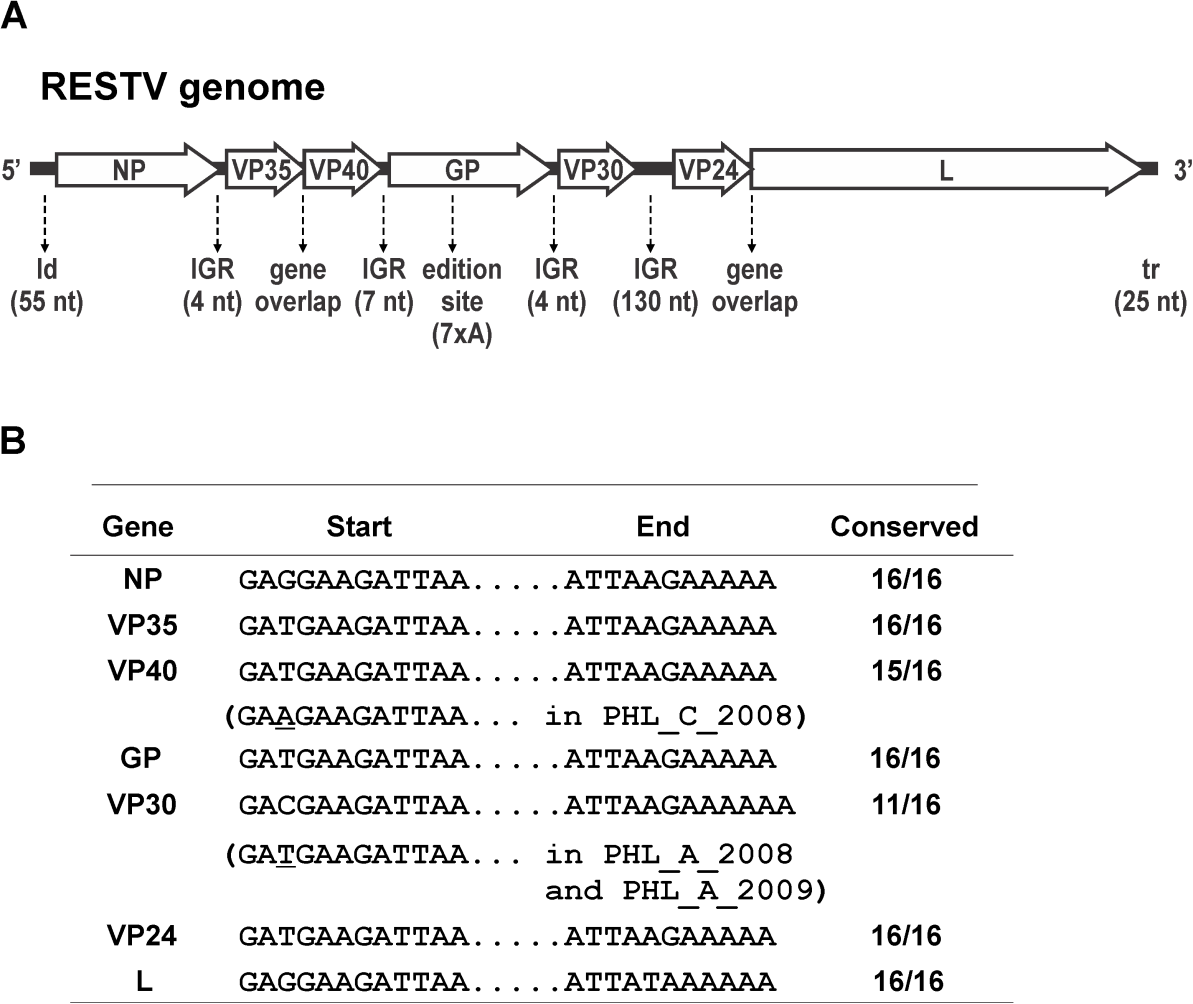
**A.** Schematic representation of RESTV genome in the viral complementary sense with the 7 encoded genes depicted in the 5′ to 3′ orientation. Major sequence features are indicated below the genome schematic with nt length in parenthesis: leader (ld), intergenic region (IGR), gene overlaps, GP edition site, and trailer (tr). **B.** Conserved sequences at the start and end of each gene are indicated. Single nucleotide changes are also indicated with asterisks.

Consistent with the genomes of all other filoviruses, the RESTV genome (depicted in viral complementary sense in Fig 3A) consists of a single-stranded, negative-sense RNA molecule of approximately 19 kb that encodes 7 genes: NP, VP35, VP40, GP, VP30, VP24, and L; these genes are transcribed sequentially from the 3′ end of the viral genome [2]. The sequence analysis of the 16 virus genomes revealed a complete conservation of the main features in the viral genome [19, 24], including the length of leader, trailer, intergenic regions, overlapped genes and the edition site in the GP gene. Moreover, the sequences corresponding to the start and end of each gene were highly conserved, while only the pig isolates have shown single nt changes in the VP40 and VP30 gene start sequences (Fig 3B).

The aa sequences of RESTV proteins were also analyzed, with special focus on changes that could potentially contribute to the virulence or pathogenicity of RESTV [2, 3, 25]. In a recent report, we compared the functionality of wild-type VP30, a viral polymerase co-factor, with a truncated version of this protein [21] that was originally found in a small percentage of EBOV genomic sequences obtained during the recent EVD outbreak in Western Africa [15, 26, 27]. Intriguingly, we also found an equivalent version of the truncated VP30 in two of the five available RESTV isolates obtained from pigs in 2008–2009. Using an NGS approach, we confirmed the presence of the mutation responsible for the VP30 truncation in the 3 isolates obtained from swine housed in Farm A, and we did not find any minor viral quasispecies carrying the wild-type VP30 sequence in these samples. Moreover, the mutation responsible for the VP30 truncation was not present as a minor quasispecies in any of the remaining isolates regardless of the animal host. Although the potential advantage of a filovirus carrying this particular mutation needs further evaluation, it has been suggested that this mutation could have arisen in EBOV as a result of virus adaption to human cells during several months of human to human transmission in the infected population during the 2013–1016 EVD outbreak [15, 27]. Based on the more abundant EBOV data, it is reasonable to hypothesize that a similar evolutionary event could have arisen if RESTV was unknowingly transmitted pig to pig for several months in swine operations in the Philippines [10, 28].

The sequences of RESTV VP35 and VP24 were also analyzed and compared with those of the human pathogenic filoviruses, since these filovirus proteins have been shown to antagonize the cellular antiviral response by inhibiting the generation of interferon (IFN) or signaling mediated by IFN [29, 30], and could be linked to apparent lack of pathogenicity of RESTV in primates other than Asian monkeys [31]. In a previous report, VP35 proteins from RESTV and EBOV were found to have similar IFN antagonist activities [32]. No significant amino acid differences were noticed in VP35 among the isolates examined here, and all sequences presented identical aa at the critical motif (RACQKSLR) in the C-terminus of this protein that has been implicated in dsRNA binding and IFN antagonist function of this protein.

Different reports have compared the IFN antagonist function of EBOV and RESTV VP24, and the difference in these proteins’ activities was localized to a central region (aa 131–146) [25, 29, 33]. All the RESTV isolates we examined exhibited 100% conservation in this area, though an aa change (L147F) was found in close proximity to the region described; this difference was only found in the two isolates obtained in 1996.

We also analyzed the aa sequences of VP35 and VP24 from the only known human-derived RESTV isolate, and found 100% identity with the corresponding RESTV proteins in the isolates obtained from non-human primates in 1989–1990 in the USA and Philippines. This conservation suggests that no evolutionary adaptation in this protein occurred after this single accidental transmission of RESTV to humans.

As in other filoviruses, RESTV GP appears to bind to the intracellular NPC1 receptor protein prior to release of the viral nucleocapsids into the cell cytoplasm [3, 34]. Although the receptor binding region has been clearly mapped mainly for EBOV GP, the analysis of the corresponding region of RESTV GP (aa ~54–201) showed a high degree of identity in all the analyzed isolates. In this regard, no genetic markers were found specifically associated with viruses isolated from primates or pigs. Similarly, the analysis of the other 6 viral proteins showed no differential markers associated with viruses isolated from primates or pigs.

**Fig 4:**
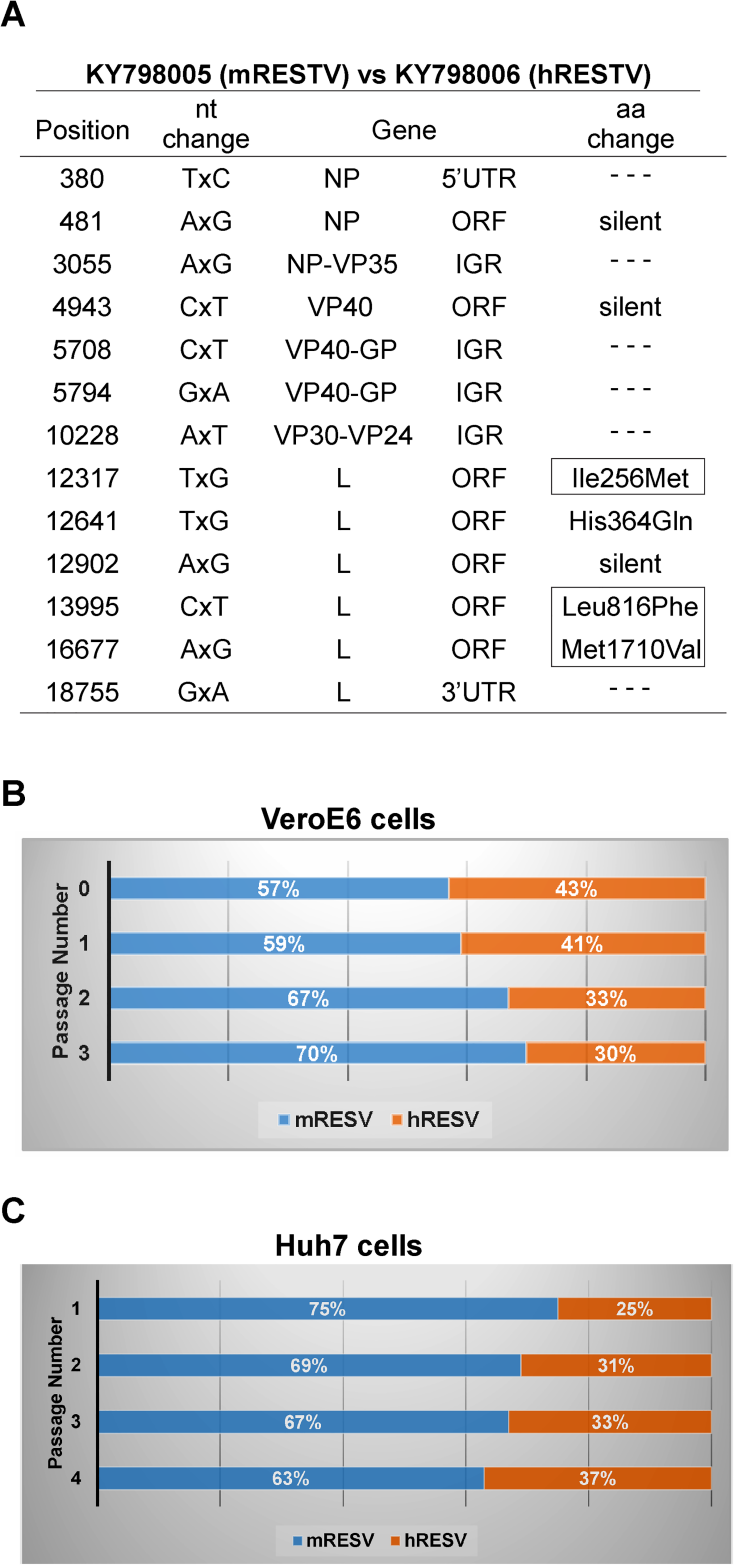
**A.** Sequence comparison of 2 viral isolates obtained during the 1989 outbreak in Virginia, USA. The positions of the nucleotide changes, gene locations, and amino acid changes are shown. Boxes are used to indicate 3 aminoacids changes in the viral polymerase that were uniquely found in the genome of the human-derived isolate (USA_VA_1989, KY798006, hRESTV). **B.** Competitive fitness assay in VeroE6 cells. Two viral isolates obtained during the 1989 outbreak in Virginia, USA (USA_VA_1989, KY798005, mRESTV and USA_VA_1989, KY798006, hRESTV) were mixed in a ~60:40% ratio and used to infect a cell monolayer in triplicate. Cell supernatants were diluted and passaged onto fresh cells 3 consecutive times. The average percentages of mRESTV and hRESTV in the supernatants are shown for each passage. **C.** Competitive fitness assay in Huh7 cells. Same viral isolates as above were mixed in an approximate ratio of 70:30% and used to infect Huh7 cells in triplicate. Cell supernatants were diluted and passaged onto fresh cells 4 consecutive times.

Finally, the sequence analysis of the viruses isolated in 1989 revealed 3 aa changes in the viral polymerase (Fig 4A) that were unique to the human-derived isolate, but were not found in any of the existing RESTV isolates from pigs or non-human primates. We compared the competitive fitness of the human-derived isolate (USA_VA_1989, KY798006, hRESTV) and the closest related isolate that was obtained from a non-human primate (USA_VA_1989, KY798005, mRESTV) in an assay for growth in a human and NHP cell line. In the competitive fitness assay performed in NHP (VeroE6) cells (Fig 4B), the hRESTV isolate represented 43% of the total virus in the original virus mix, but decreased to 41%, 33% and 30% of the total virus by passages 1, 2 and 3, respectively. The opposite trend was observed when the virusese were passed on the human cells, with the hRESTV isolate increasing in proportion from 25% at passage 1, to 37% after 4 passages (Fig 4C).

## Conclusions

While the natural reservoirs of filoviruses are still unknown, molecular and/or serological evidence have revealed the presence of EBOV, MARV, and RAVV in African fruit bats, and LLOV in insectivorous bats from Spain [13, 35–37], Moreover, the isolation of MARV and RAVV from African fruit bats added further support to the hypothesis that bats are a main reservoir of filoviruses in nature [38]. Similarly, RESTV was also detected by molecular or serological methods in fruit bats from the Philippines, Bangladesh, and China [39–42].

In agreement with previous studies [12, 25, 28], our phylogenetic analysis suggests the occurrence of multiple independent introductions of RESTV from the still unknown natural reservoir into the breeding facilities of non-human primates and the swine farming operations in the Philippines. In future studies, the analysis of complete filovirus genomes obtained from potential natural reservoirs will significantly improve the understanding of the underlying mechanisms of an outbreak, especially the initial spillover events into other host species.

Furthermore, based on the sequence analysis of the viral proteins, no major differences in the *in-vivo* phenotype are expected for the RESTV isolates examined in this study, although further experimentation in animal models will be needed to confirm this hypothesis.

Finally, we presented in this report the first experimental study on the only available RESTV isolated from a human sample. The sequence analysis of this isolate revealed only minor sequence changes when compared with another closely related virus. Interestingly, these 2 viruses exhibited an opposite fitness trend when grown in NHP or in a human cell line. Although modest, the fitness difference is consistent with their isolation source.

## Supporting information

S1 Fig Bayesian analysis with sequence outgroup. (TIF)

S2 Fig Phylogenetic analysis using Maximum-Likelihood. (TIF)

S1 Table NGS details for each isolate. (PDF)

S2 Table Additional NGS details of fitness assay. (PDF)
