# Supplementary material for: Insights into Reston virus spillovers and adaption from virus whole genome sequences
Source: PLoS One. 2017 May 25;12(5):e0178224. doi: 10.1371/journal.pone.0178224 (PMC5444788; doi:10.1371/journal.pone.0178224)
Supplement: S1 Table — (PDF) [file pone.0178224.s003.pdf]

Isolate name	Mapped reads	Consensus length [a]	Ambiguous [b] 5'end // 3'end	Percentage of Ref. Seq. [c]	Average coverage
USA_PA_1989 (813159)	2,652,487	18,891	N/A	100 %	20,342
USA_VA_1989 (813168)	215,727	18,881	1 // 9	> 99 %	1,659
USA_VA_1989 (811952)	382,376	18,883	1 // 7	> 99 %	2,934
ITA_1992 (806679)	196,202	18,872	12 // 7	> 99 %	1,523
PHL_1992 (806676)	48,983	18,875	1 // 15	> 99 %	361
USA_TX_1996 (807334)	119,814	18,879	1 // 11	> 99 %	887
PHL_A_2008 (811411)	628,462	18,879	1 // 7 [d]	> 99 %	4,729
PHL_A_2008 (811412)	204,006	18,868	8 // 11 [d]	> 99 %	1,514
PHL_A_2009 (813161)	217,371	18,879	1 // 7 [d]	> 99 %	1,635

[a] Non-ambiguous sequence
[b] Ambiguous or missing terminal nt
[c] Reference sequence: NC_004161 is 18,891 nt long
[d] VP24 5' UTR is 4 nt shorter than NC_004161
