# Supplementary material for: Insights into Reston virus spillovers and adaption from virus whole genome sequences
Source: PLoS One. 2017 May 25;12(5):e0178224. doi: 10.1371/journal.pone.0178224 (PMC5444788; doi:10.1371/journal.pone.0178224)
Supplement: S2 Table — (PDF) [file pone.0178224.s004.pdf]

Pass.No.	1-A			1-B			1-C			
Position	Count	Coverage	Freq. %	Count	Coverage	Freq. %	Count	Coverage	Freq. %	Avg.
380	1984	8112	24	2603	9860	26	2092	8415	25	**25**
481	2096	8571	24	2688	10529	26	2217	8587	26	
3055	1795	7017	26	2162	8269	26	1787	6602	27	
4943	2719	11674	23	3397	13259	26	2478	9873	25	
5708	2001	9536	21	2641	11224	24	1952	8230	24	
5794	1246	5767	22	1666	7198	23	1271	5602	23	
10228	2347	9849	24	3019	11747	26	2210	8831	25	
12317	1212	4408	27	1607	5745	28	1348	4920	27	
12641	997	3868	26	1403	5154	27	1204	4380	27	
12902	2305	9184	25	3051	11142	27	2446	9319	26	
13995	2080	9048	23	2839	11111	26	2068	8394	25	
16677	2183	8390	26	2861	10280	28	2345	8377	28	
18755	183	977	19	240	1154	21	215	915	23	

Pass.No.	2-A			1-B			2-C			
Position	Count	Coverage	Freq. %	Count	Coverage	Freq. %	Count	Coverage	Freq. %	Avg.
380	1169	3997	29	1808	5723	32	5529	17672	31	**31**
481	1235	4216	29	1828	5763	32	5546	18119	31	
3055	1010	3210	31	1429	4337	33	4348	14158	31	
4943	2201	7725	28	2332	7327	32	6286	21157	30	
5708	1145	4531	25	1487	5128	29	5415	19394	28	
5794	595	2417	25	986	3383	29	3592	12653	28	
10228	1526	5483	28	1723	5450	32	5767	19320	30	
12317	563	1710	33	1155	3186	36	3140	9548	33	
12641	533	1644	32	851	2515	34	3001	9038	33	
12902	1454	4882	30	2018	6197	33	5421	17408	31	
13995	1541	5383	29	1698	5668	30	4969	16991	29	
16677	1269	4004	32	1999	5867	34	5331	16244	33	
18755	74	254	29	134	383	35	678	2395	28	

Pass.No.	3-A			3-B			3-C			
Position	Count	Coverage	Freq. %	Count	Coverage	Freq. %	Count	Coverage	Freq. %	Avg.
380	2834	8574	33	6737	19842	34	2739	8368	33	**33**
481	3011	9081	33	6988	20649	34	2756	8432	33	
3055	1579	4758	33	3861	11127	35	1462	4404	33	
4943	2089	6432	32	4930	14827	33	2059	6552	31	
5708	2272	7944	29	5951	18985	31	2116	7105	30	
5794	1534	5311	29	3980	12541	32	1432	4870	29	
10228	2758	8736	32	6675	19667	34	2493	7994	31	
12317	1460	4312	34	3514	9706	36	1386	3943	35	
12641	1477	4195	35	3312	9128	36	1154	3298	35	
12902	2473	7493	33	5722	15665	37	2369	7010	34	
13995	2081	6621	31	5218	15036	35	1947	6013	32	
16677	2596	7569	34	6052	16594	36	2632	7402	36	
18755	530	1800	29	1292	3945	33	322	1045	31	

Pass.No.	4-A			4-B			4-C			
Position	Count	Coverage	Freq. %	Count	Coverage	Freq. %	Count	Coverage	Freq. %	Avg.
380	5534	15180	36	6100	15108	40	8031	22780	35	**37**
481	5561	15189	37	5514	13850	40	7456	20746	36	
3055	2751	7488	37	2694	6409	42	3583	9677	37	
4943	3761	10755	35	4327	11150	39	5914	17270	34	
5708	4023	12453	32	3509	9473	37	5816	18214	32	
5794	2660	8097	33	2533	6837	37	3660	11673	31	
10228	4238	12480	34	3793	9683	39	6408	18452	35	
12317	2178	5759	38	2073	4823	43	2848	7348	39	
12641	1925	4996	39	1909	4336	44	2512	6405	39	
12902	3891	10324	38	4218	10000	42	5744	15387	37	
13995	3019	8635	35	3177	7941	40	4397	12929	34	
16677	3606	9472	38	4302	10086	43	5420	14141	38	
18755	430	1296	33	626	1687	37	463	1539	30	
